# *nab*-Paclitaxel Plus Durvalumab in Patients With Previously Treated Advanced Stage Non-small Cell Lung Cancer (ABOUND.2L+)

**DOI:** 10.3389/fonc.2020.569715

**Published:** 2021-02-11

**Authors:** Daniel Morgensztern, Manuel Cobo Dols, Santiago Ponce Aix, Pieter E. Postmus, Jaafar Bennouna, Jürgen R. Fischer, Oscar Juan-Vidal, David J. Stewart, Andrea Ardizzoni, Rafia Bhore, Marianne Wolfsteiner, Martin Reck, Denis Talbot, Ramaswamy Govindan, Teng Jin Ong

**Affiliations:** ^1^Washington University School of Medicine, St Louis, MO, United States; ^2^Hospital Universitario Málaga Regional, Instituto de Investigación Biomédica de Málaga (IBIMA), Málaga, Spain; ^3^Unidad de Investigación Clínica de Cáncer Pulmón H12O-CNIO, Madrid, Spain; ^4^Clatterbridge Cancer Center, Liverpool, United Kingdom; ^5^Centre René Gauducheau Centre de Lutte Contre Le Cancer Nantes Atlantique, Nantes, France; ^6^Lungenklinik Löwenstein gGmbH, Löwenstein, Germany; ^7^Universitari i Politécnic La Fe, Valencia, Spain; ^8^Ottawa Hospital, Ottawa, ON, Canada; ^9^Azienda Ospedaliero Universitaria Di Bologna—Policlinico S. Orsola-Malpighi, Bologna, Italy; ^10^Bristol Myers Squibb, Princeton, NJ, United States; ^11^Pharmaceutical Research Associates Inc. (PRA) Health Sciences, Lenexa, KS, United States; ^12^LungenClinic, Grosshansdorf, Germany; ^13^Churchill Hospital, Oxford, United Kingdom

**Keywords:** advanced stage non-small cell lung cancer, durvalumab, immune checkpoint blocker plus chemotherapy, *nab*-paclitaxel, second-line therapy

## Abstract

**Background:** The standard therapy for advanced stage non-small cell lung cancer (NSCLC) with no actionable gene alterations is a platinum-based chemotherapy doublet and immune checkpoint blocker (ICB), either concurrently or sequentially, followed by docetaxel at the time of tumor progression. However, more effective treatments are needed. We evaluated the *nab*-paclitaxel and durvalumab combination in patients with previously treated advanced stage NSCLC.

**Methods:** Patients with advanced stage NSCLC previously treated with one line of platinum-based doublet with or without an ICB and no activating *EGFR* mutations or *ALK* translocations received *nab*-paclitaxel 100 mg/m^2^ (days 1 and 8) plus durvalumab 1,125 mg (day 15) every 21 days. The primary endpoint was progression-free survival (PFS). Key secondary endpoints included overall survival (OS) and safety.

**Results:** Between February 2016 and December 2016, 79 patients were enrolled. The median age was 63 years. Most patients were males (68.4%), had non-squamous histology (69.6%), and had no prior ICB treatment (88.6%). The median PFS was 4.5 months; median OS was 10.1 months. A *post hoc* analysis of survival by prior ICB treatment revealed a median PFS and OS of 4.4 and 9.9 months, respectively, in ICB-naive patients and 6.9 months and not estimable, respectively, in patients previously treated with ICB. The most common treatment-emergent adverse events were asthenia (46.2%) and diarrhea (34.6%); four treatment-related deaths (5.1%) occurred.

**Conclusions:** The *nab*-paclitaxel and durvalumab combination is feasible and demonstrated antitumor activity without new safety signals. Additional studies using taxanes and ICB in patients with previously treated NSCLC are warranted.

**Clinical Trial Registration:** ClinicalTrials.gov registration (NCT02250326).

**EudraCT number:** 2014-001105-41

## Introduction

The standard initial therapy for patients with advanced stage non-small cell lung cancer (NSCLC) and no actionable gene alterations includes platinum-based chemotherapy doublet and immune checkpoint blockers (ICB) either sequentially or concurrently (1, 2). For patients previously treated with chemotherapy alone, monoclonal antibodies against programmed death-1 (PD-1) or its ligand (PD-L1) are associated with improved overall survival (OS) when compared to docetaxel in the second-line setting, although prolonged benefit is observed only in a small percentage of patients (3–6). For those already treated with both chemotherapy and ICB, docetaxel with or without ramucirumab remains the standard option (7). Response rates and survival, however, remain poor for the majority of patients treated with second-line ICB monotherapy or docetaxel, indicating the need for new treatment options.

Nanoparticle albumin-bound paclitaxel (*nab*-paclitaxel), a cremophor-free formulation that can be administered without dexamethasone premedication (8), is one of the recommended drugs for locally advanced or metastatic NSCLC in combination with carboplatin, with or without pembrolizumab, in the first-line setting for patients who are not candidates for curative surgery or radiation therapy (2, 9). Single-agent *nab*-paclitaxel has been associated with promising results in previously treated patients with metastatic NSCLC (10, 11) and better tolerability compared with docetaxel in a randomized clinical trial for patients with metastatic breast cancer (12).

Durvalumab is a human IgG1 monoclonal antibody against PD-L1, approved as consolidation therapy after chemoradiation in patients with stage III NSCLC (13). In patients with advanced stage NSCLC, single-agent durvalumab is associated with similar activity and safety profiles when compared with other ICBs (14).

Based on both preclinical (15) and clinical (16–18) studies demonstrating a benefit from concurrent chemotherapy and ICB in NSCLC, we postulated that the same principles may apply to patients treated with *nab*-paclitaxel after progression on platinum-based chemotherapy with or without ICB.

ABOUND.2L+ was a randomized clinical trial comparing *nab*-paclitaxel alone or in combination with CC-486, an oral formulation of azacitidine (19). The study showed no benefit from the addition of azacitidine to *nab*-paclitaxel in the randomized cohorts of the study, although single-agent *nab*-paclitaxel was associated with a tolerable safety profile and promising outcomes, including response rates, median progression-free survival (PFS), and median OS of 16.3%, 4.2, and 17.0 months, respectively. Here we present the results of the third arm of the study evaluating the combination of *nab*-paclitaxel and durvalumab, which was non-randomized and added as an amendment.

## Materials and Methods

### Patients

Eligible patients were 18 years of age or older and had histologically or cytologically confirmed advanced stage NSCLC, radiologically documented measurable disease by Response Evaluation Criteria In Solid Tumors (RECIST) 1.1, an Eastern Cooperative Oncology Group performance status 0 or 1, adequate hematologic, renal, and hepatic function, and no other current active malignancy requiring anticancer therapy. One prior line of platinum-based chemotherapy regimen for metastatic or recurrent disease was allowed, with the exception of taxanes, which were allowed only if used in the adjuvant setting more than 12 months prior to enrollment into the trial. Prior use of ICBs, either as a component of the first-line therapy or in the second line, was allowed. Key exclusion criteria included known activating *EGFR* mutations or *ALK* translocations, peripheral neuropathy grade 2 or higher by the National Cancer Institute Common Terminology Criteria for Adverse Events (NCI-CTCAE) version 4.0, active or prior documented autoimmune or inflammatory disorder, use of systemic immunosuppressive therapy within 14 days from starting durvalumab except for corticosteroids, at doses up to 10 mg per day of prednisone or its equivalent, and brain metastases unless asymptomatic and clinically stable for at least 8 weeks following completion of therapy.

The study was approved by the institutional review board or independent ethics committee at participating sites and conducted in accordance with the principles of good clinical practice and the Declaration of Helsinki. All patients provided written informed consent prior to treatment initiation.

### Study Design

This was an open-label phase II study. Initially, patients were randomized 1:1 to receive *nab*-paclitaxel 100 mg/m^2^ on days 8 and 15 plus CC-486 200 mg on days 1 to 14 or *nab*-paclitaxel 100 mg/m^2^ alone on days 1 and 8 of each 21-day cycle (19). After enrollment for the *nab*-paclitaxel alone and *nab*-paclitaxel plus CC-486 arms was completed, the protocol was amended to include a third arm, which enrolled patients with advanced stage non-squamous or squamous NSCLC and one prior platinum-based chemotherapy. Patients were assigned to this arm and received *nab*-paclitaxel 100 mg/m^2^ infused over 30 min on days 1 and 8 plus durvalumab 1,125 mg infused over 1 h on day 15, with the cycles repeating every 21 days. Hence, randomization did not occur between the *nab*-paclitaxel plus durvalumab and *nab*-paclitaxel alone arms. Treatment was continued until documented tumor progression, unacceptable toxicity, withdrawal of consent, lost to follow-up, or death.

### Endpoints and Assessments

The primary endpoint was the duration of PFS in the intent-to-treat (ITT) population, defined as the time from date of treatment initiation to the date of disease progression, based on investigator assessment using RECIST version 1.1, or death from any cause. Secondary endpoints included OS, defined as the time between the first treatment and death from any cause, overall response rate (ORR), disease control rate (DCR), and safety. Imaging studies with computer tomography scans were performed at baseline and every 42 days until treatment discontinuation. All patients who received at least one treatment dose underwent safety analysis, with documentation of treatment-emergent adverse events (TEAEs) graded based on NCI-CTCAE version 4.0.

### Statistical Analyses

The median PFS and median OS were estimated using the Kaplan-Meier estimates with corresponding two-sided 95% confidence intervals (CI). The sample size estimation was based on the expected median PFS of 4.25 months for *nab*-paclitaxel plus durvalumab and 2.5 months for *nab*-paclitaxel alone based on historical data with docetaxel alone (7, 20, 21).

In the randomized part of the trial, it was estimated that a total of 160 patients would be needed to observe 120 PFS events, which would have provided 80% power to detect a hazard ratio of 0.60 using a one-sided test at the 2.5% level of significance. After enrollment in the *nab*-paclitaxel plus CC-486 and *nab*-paclitaxel monotherapy arms was completed (each arm had reached a total of approximately 80 patients), all patients were assigned to the *nab*-paclitaxel plus durvalumab arm until approximately 80 patients were enrolled in that arm. The statistical assumptions used were identical to the *nab*-paclitaxel plus CC-486 arm. An interim analysis for PFS comparing the *nab*-paclitaxel plus durvalumab and *nab*-paclitaxel monotherapy arms was conducted when approximately 30 PFS events were observed in the *nab*-paclitaxel plus durvalumab combination arm.

## Results

### Patients

**Figure 1:**
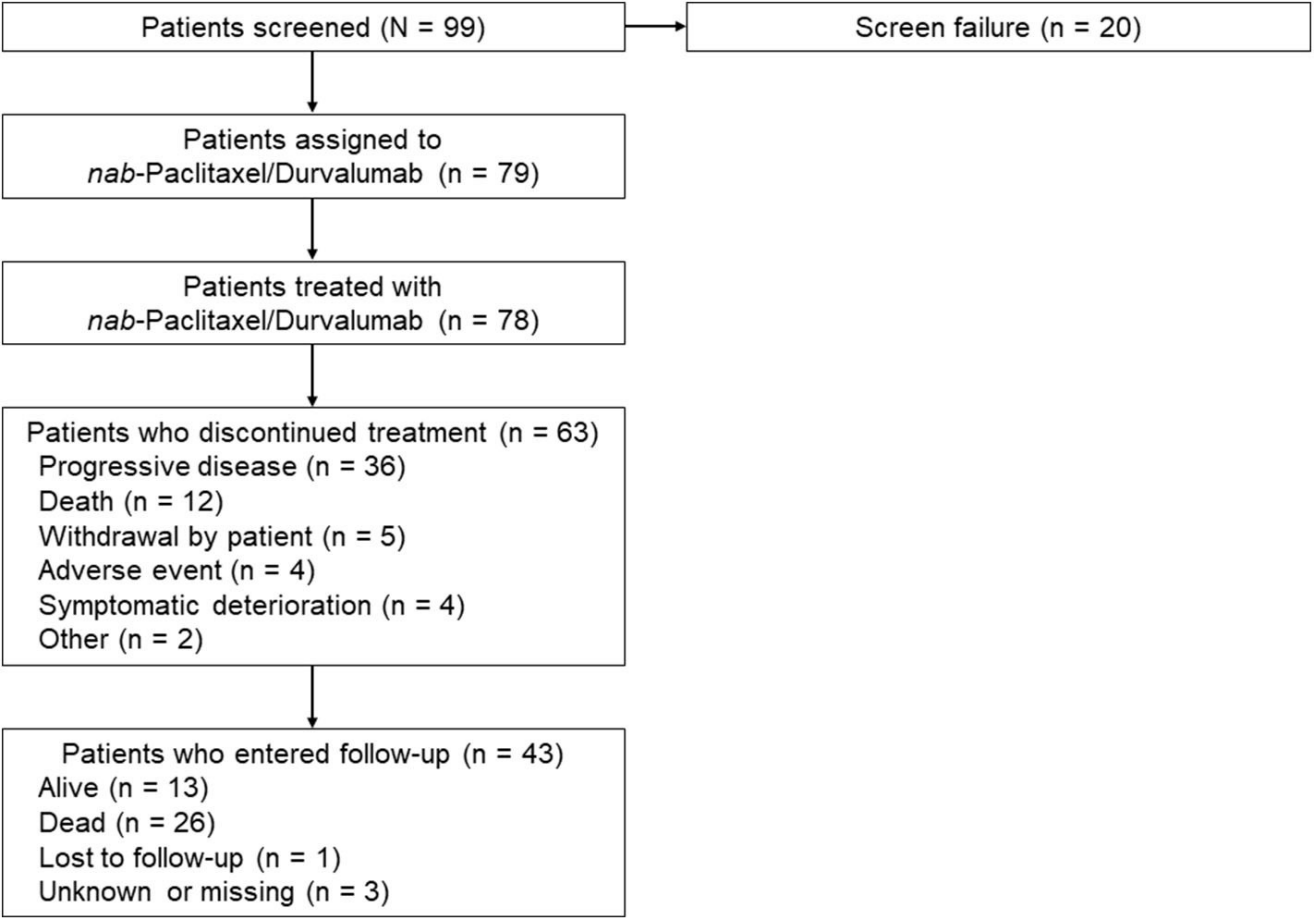
CONSORT diagram.

Between September 2016 and December 2016, 99 patients were screened and 79 were enrolled into the study (Figure 1). The median age was 63 years (range 29–84 years); most patient were males (68.4%) and had non-squamous histology (69.6%) and no prior use of ICB (88.6%) (Table 1). Prior chemotherapies included a platinum (97.5%), pemetrexed (50.6%), vinorelbine (25.3%), and gemcitabine (26.6%). The median duration of prior platinum plus pemetrexed (39 patients) was 10.4 weeks (range 1.6–43.4 weeks). In total, nine patients (11.4%) received prior ICB, which was their most immediate prior line of therapy. Among these nine patients, six received nivolumab (66.7%), two received pembrolizumab (22.2%), and one received avelumab (11.1%), with the latter in the first-line setting. Of these nine patients, eight (88.9%) received prior ICB monotherapy. The remaining patient (11.1%) received prior combination therapy with ICB and carboplatin. One patient did not receive the study treatment. In total, 63 patients (80.8%) discontinued treatment due to progressive disease (36 [46.2%]), death (12 [15.4%]), patient withdrawal (5 [6.4%]), clinical progression (4 [5.1%]), adverse events (4 [5.1%]), and other reasons (2 [2.6%]). The adverse events (AEs) leading to *nab*-paclitaxel and durvalumab discontinuation were pneumonitis, urinary tract infection, and *Pneumocystis jirovecii* pneumonia (one patient each) and increased white blood cell count, abnormal liver function, localized edema, and peripheral edema (one patient). The median follow-up for survival was 12.9 months.

**Table 1:** Demographic and baseline clinical characteristics.

**Characteristic**	***nab*-Paclitaxel + Durvalumab (*N* = 79)**
Age, years	
Mean (SD)	62.7 (10.74)
Median (range)	63.0 (29–84)
Male sex, number (%)	54 (68.4%)
ECOG performance status, number (%)	
0	18 (22.8%)
1	61 (77.2%)
Stage IV disease, number (%)	75 (94.9%)
Histology	
Squamous	23 (29.1%)
Non-squamous	55 (69.6%)
Not specified	1 (1.3%)
Prior therapy	
No prior ICB	70 (88.6%)
Prior ICB	9 (11.4%)^a^

a *One patient with prior ICB treatment received first-line avelumab without platinum-based chemotherapy*.

*ECOG, Eastern Cooperative Oncology Group; ICB, immune checkpoint blocker*.

### Efficacy

**Figure 2:**
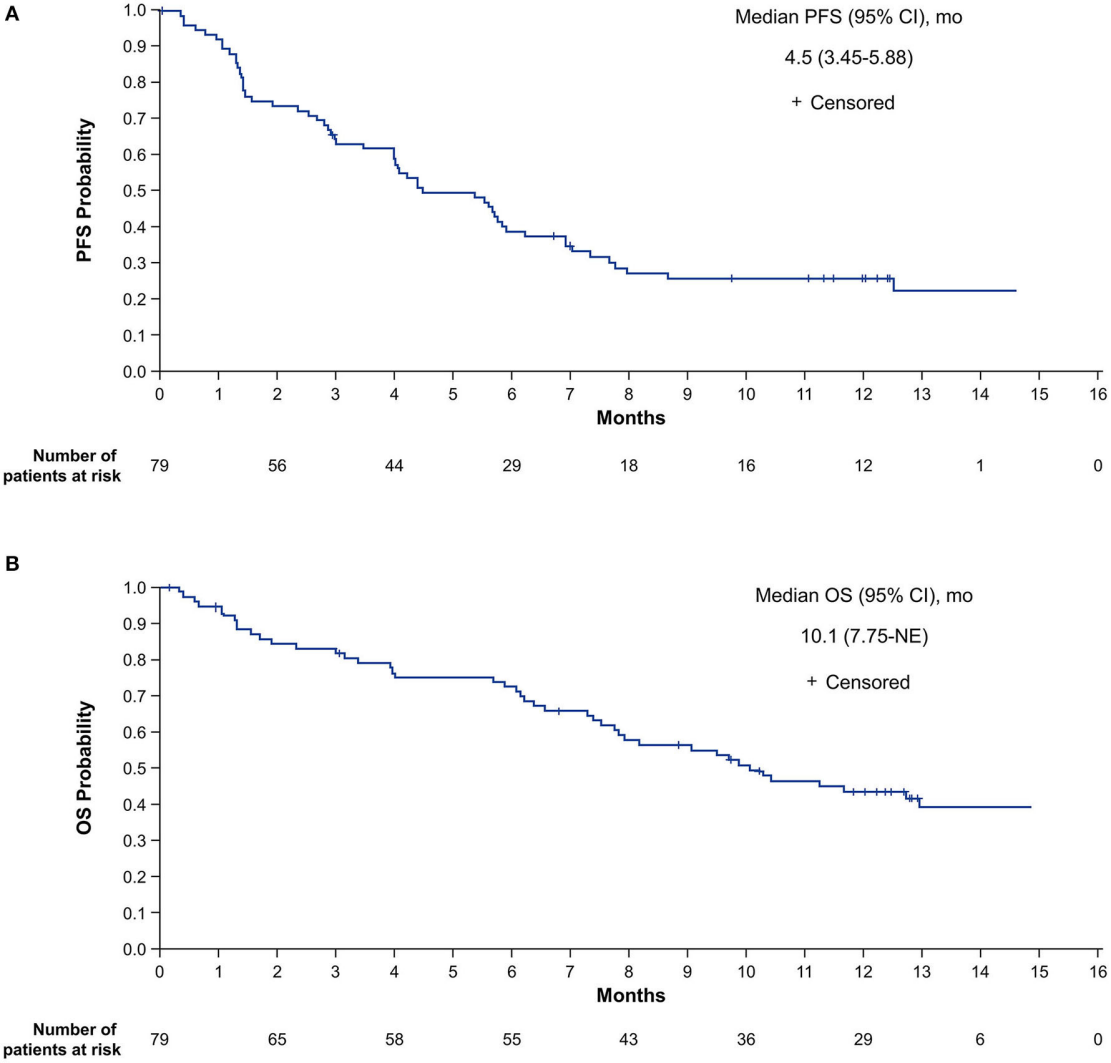
Investigator-assessed PFS **(A)** and OS **(B)** in the intent-to-treat population. NE, not estimable; OS, overall survival; PFS, progression-free survival.

For the primary analysis of investigator-assessed PFS in the ITT population, 56 patients (70.9%) had progressive disease (PD) or died. The median PFS was 4.5 months (95% CI, 3.5–5.9 months), with an estimated PFS rate at 12 months of 25.7% (95% CI, 16.3–36.2%; Figure 2A).

For the OS analysis in the ITT population, 44 patients (55.7%) had died. The median OS was 10.1 months (95% CI, 7.8 months-not estimable [NE]), with estimated survival at 12 months of 43.8% (95% CI, 32.3–54.7%; Figure 2B).

The ORR was 27.8% (95% CI, 18.3–39.1%), with complete response (CR) in one patient (1.3%) and partial response (PR) in 21 patients (26.6%). The DCR was 70.9% (95% CI, 59.6–80.6%), with 34 patients (43.0%) achieving stable disease (SD).

**Figure 3:**
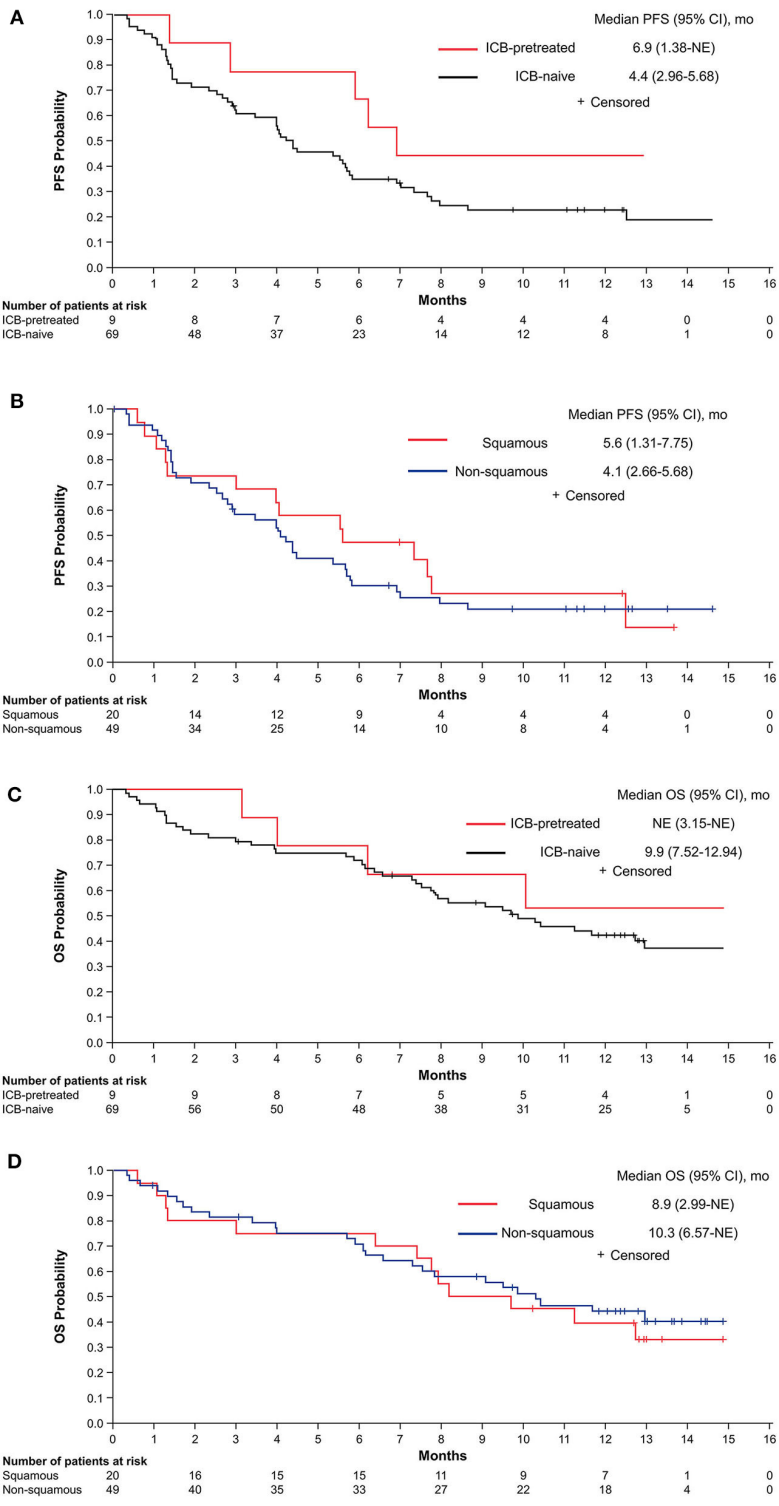
PFS by ICB treatment status **(A)** and in ICB-naive patients according to histology **(B)** and OS by ICB treatment status **(C)** and in ICB-naive patients according to histology **(D)**. ICB, immune checkpoint blocker; NE, not estimable; OS, overall survival; PFS, progression-free survival.

Due to the heterogeneity of the patient population, a *post hoc* analysis was performed to evaluate outcomes according to prior ICB treatment and histology in the 78 patients with known histology. The median PFS was 4.4 months (95% CI, 2.96–5.68 months) in ICB-naive patients and 6.9 months (95% CI, 1.38 months-NE) in patients previously treated with ICB (Figure 3A). Among ICB-naive patients, the median PFS was 5.6 months (95% CI, 1.3–7.8 months) in those with squamous histology and 4.1 months (2.7–5.7 months) in those with non-squamous histology, with corresponding 12-month PFS of 27.1% (95% CI, 9.0–49.2%) and 20.9% (95% CI, 10.6–33.6%), respectively (Figure 3B). The median OS was 9.9 months (95% CI, 7.52–12.94 months) in ICB-naive patients and NE in those previously treated with ICB (Figure 3C). Among ICB-naive patients, the median OS for squamous and non-squamous histologies was 8.9 months (95% CI, 2.99 months-NE) and 10.3 months (95% CI, 6.57 months-NE), respectively (Figure 3D).

**Figure 4:**
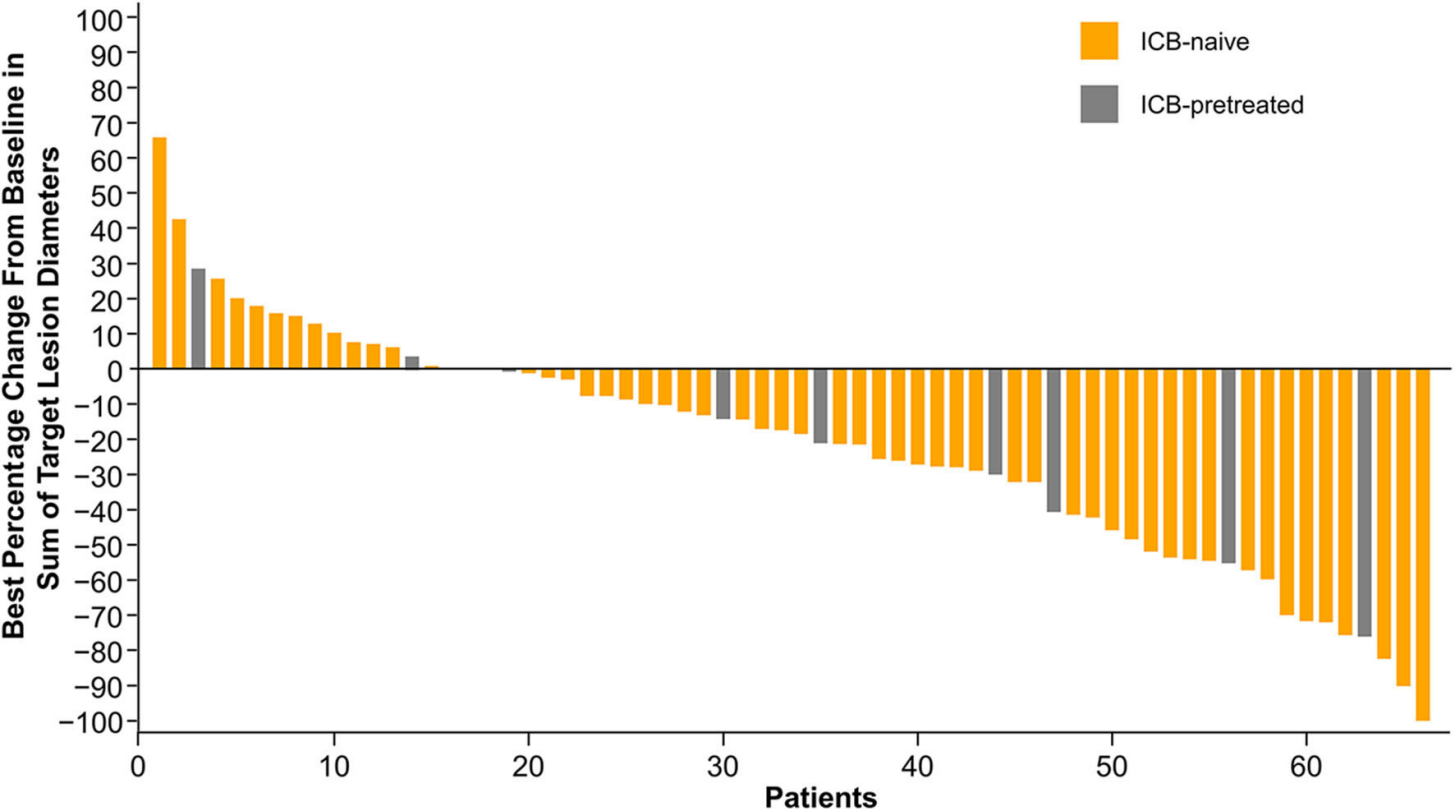
Percentage of tumor change from baseline. ICB, immune checkpoint blocker.

The median percentage change from baseline in sum of diameters of target lesions was −17.3% (range −100.0 to +65.4%) for ICB-naive patients and −21.4% (range −76.2 to +28.1%) for those previously treated with ICB (Figure 4). Among ICB-naive patients, one achieved CR (1.4%), 17 achieved PR (24.6%), and 30 had SD (43.5%) for a DCR of 69.6%. Of the remaining patients, 10 had PD (14.5%) and 11 (15.9%) had no post-treatment response assessment. Among patients previously treated with ICB, four achieved PR (44.4%), four achieved SD (44.4%), and one had PD (11.1%).

### Treatment Exposure

The median number of cycles and treatment duration were 7 (range 1–21) and 24.4 weeks (range 1.4–66.1 weeks), respectively. The median cumulative doses of *nab*-paclitaxel and durvalumab were 1,250 mg/m^2^ and 6,750 mg, respectively. Dose reductions for *nab*-paclitaxel due to toxicity occurred in 11 patients (14.1%); per protocol, durvalumab dose reductions were not allowed. Dose delays of *nab*-paclitaxel and durvalumab occurred in 39 patients (50%) and 24 patients (30.8%), respectively.

### Safety

All patients developed at least one TEAE, with grade 3 or 4 TEAEs occurring in 43 patients (55.1%) (Table 2). The most common TEAEs of any grade were asthenia (46.2%), diarrhea (34.6%), and decreased appetite (33.3%), while the most common grade 3 or 4 TEAEs were asthenia (12.8%), dyspnea (7.7%), and pneumonia (7.7%). Peripheral neuropathy was seen in 29 patients (37.2%), of which 3 (3.8%) were grade 3 or 4.

**Table 2:** Treatment-emergent adverse events occurring in at least 15% of patients.

**TEAE, *n* (%)**	***nab*****-Paclitaxel** **+** **Durvalumab (***n* = **78)**	
	**Any grade**	**Grade 3 or 4**
Any event	78 (100.0%)	43 (55.1%)
Asthenia	36 (46.2%)	10 (12.8%)
Diarrhea	27 (34.6%)	1 (1.3%)
Decreased appetite	26 (33.3%)	1 (1.3%)
Alopecia	25 (32.1%)	0
Anemia	24 (30.8%)	4 (5.1%)
Peripheral neuropathy	29 (37.2%)	3 (3.8%)
Fatigue	22 (28.2%)	2 (2.6%)
Upper respiratory tract infection	22 (28.2%)	2 (2.6%)
Constipation	20 (25.6%)	0
Dyspnea	20 (25.6%)	6 (7.7%)
Nausea	19 (24.4%)	0
Cough	19 (24.4%)	0
Pyrexia	15 (19.2%)	0
Neutropenia	14 (17.9%)	5 (6.4%)
Lower respiratory tract infection	12 (15.4%)	1 (1.3%)

*TEAE, treatment-emergent adverse event*.

Immune-related TEAEs of grade 3 or 4 were observed in seven patients (9.0%). The grade 3 or 4 immune-related TEAEs were diarrhea (1 [1.3%]), rash (2 [2.6%]), pneumonitis (1 [1.3%]), and adrenal insufficiency (3 [3.8%]). Among the nine patients who received prior ICB, immune-related TEAEs of grade 3 or 4 were observed in two patients (22.2%). The grade 3 or 4 immune-related TEAEs were adrenal insufficiency and rash (1 patient [11.1%] each). Other AEs of interest included grade 1 or 2 dermatitis (10.3%) and thyroid dysfunction (hypothyroidism, 6.4%; hyperthyroidism, 2.6%; thyroiditis, 1.3%).

Overall, four patients (5.1%) experienced a grade 5 TEAE suspected to be treatment related. The specific grade 5 treatment-related TEAEs were pneumonitis, pulmonary hemorrhage, *Pneumocystis jirovecii* pneumonia, and clinical deterioration.

## Discussion

The median PFS of 4.5 months exceeded the pre-specified threshold, and the response rate of 27.8% is higher than previously described in patients treated with either docetaxel or ICB monotherapy (3–6).

There are increasing data suggesting that the efficacy of conventional chemotherapy drugs relies not only on their cytotoxic effects, but also on the ability to stimulate the immune system. In the case of paclitaxel, there are many postulated mechanisms for its immunostimulatory effects in addition to tumor debulking in case of effective cytotoxic activity, with reduction of the systemic immunosuppression caused by malignant cells. Paclitaxel induces immunogenic cell death through increased chromosomal content, which causes endoplasmic stress response and calreticulin exposure, stimulates toll-like receptor 4 increasing T cell priming by dendritic cells, and depletes myeloid derived suppressor cells (22, 23). Paclitaxel may also increase the antigenicity of cancer cells by stimulating their production of interferon-β, leading to increasing MHC class I expression (24, 25). Another mechanism is the sensitization to cytotoxic T lymphocytes by upregulating mannose-6-phosphate receptors on tumor cells, which increases the permeability of the membrane to granzyme B, leading to cancer cell death independent from perforin (26). Since *nab*-paclitaxel does not require the use of premedication with corticosteroids, it may be a better partner for combination with ICB when compared with other taxanes since, at least in patients treated with single-agent ICB, use of corticosteroids at doses of 10 mg or higher has been associated with worse outcomes compared with no use within 30 days (27).

There are limited data on the combination of taxanes and ICB without platinum in patients with previously treated NSCLC. In a small phase Ib study evaluating the combination of chemotherapy and nivolumab 10 mg/kg every 3 weeks, there were six patients previously treated with platinum-doublets who were enrolled into the docetaxel arm (28). One patient (16.5%) responded to the treatment, and the median PFS was 3.1 months. All patients developed grade 3 or 4 AEs, which were mostly hematologic.

In our study, the combination of *nab*-paclitaxel plus durvalumab was generally well-tolerated; however, the 4 grade 5 treatment-related TEAEs were an unexpected finding. Patients in the *nab*-paclitaxel plus durvalumab arm received more treatment cycles and a greater cumulative dose of *nab*-paclitaxel compared with those who received *nab*-paclitaxel with or without CC-486 in the randomized portion of this trial (19). Therefore, it is reasonable to speculate that the grade 5 treatment-related TEAEs were due, at least in part, to a greater treatment exposure with second-line combination therapy. Although there were no grade 5 AEs reported with second-line pembrolizumab plus docetaxel in the phase II PROLUNG trial (29), that study accrued patients considerably younger than those treated with *nab*-paclitaxel plus durvalumab in the current study (mean, 50.1 vs. 62.7 years).

Our study has several limitations. The durvalumab arm started enrollment after the completion of the randomized *nab*-paclitaxel with or without CC-486, precluding a more reliable comparison to single-agent *nab*-paclitaxel, and the increased use of pembrolizumab or atezolizumab in the first-line setting decreased the number of ICB-naive patients eligible for the *nab*-paclitaxel plus durvalumab combination in the clinical setting (16–18). Furthermore, we did not collect data on PD-L1 status of the tumors or genetic biomarkers, which are known predictors for response to ICBs in previously untreated patients (5, 30), although the role is not clear in patients with resistance to ICBs.

Nevertheless, despite these limitations, our study provides the initial data on the use of *nab*-paclitaxel plus durvalumab after progression on ICB, a setting with increased relevance since the trial was designed. Despite the multiple ongoing studies evaluating combinations involving antibodies against PD-1 or PD-L1 with other immunostimulatory antibodies, antiangiogenic agents and targeted drugs (31–33), none has an established role in NSCLC patients previously treated with ICBs. Although the number of patients previously treated with ICB in our study was small, the preliminary results are promising, with all but one patient achieving tumor control and a prolonged benefit observed in four of the nine patients.

Since there are limited data on the efficacy of docetaxel after tumor progression on ICB and we cannot clearly separate the effects of *nab*-paclitaxel and durvalumab, this question could only be addressed in a randomized clinical trial comparing a taxane, either docetaxel or *nab*-paclitaxel, alone or in combination with an ICB.

## Data Availability Statement

The datasets presented in this article are not readily available. Data requests may be submitted to Celgene, A Bristol Myers Squibb Company, at: https://vivli.org/ourmember/celgene/ and must include a description of the research proposal.

## Ethics Statement

Written informed consent for participation was not required for this study in accordance with the national legislation and the institutional requirements.

## Author Contributions

All authors satisfied the following criteria: contributed to the conception or design of the research or the acquisition, analysis, or interpretation of data for the research, drafted the manuscript or critically revised it for important intellectual content, gave final approval of the version to be published, agreed to be accountable for all aspects of the research in ensuring that questions related to the accuracy or integrity of any part of the work are appropriately investigated and resolved.

## Conflict of Interest

DM has been an advisory board member for AbbVie, Bristol Myers Squibb, PharmaMar, Takeda, Gilead, and Boehringer Ingelheim. PEP has been an advisory board member for AstraZeneca, Boehringer Ingelheim, Bristol Myers Squibb, Celgene (a Bristol Myers Squibb Company), Clovis, Janssen, MSD, and Roche. JB has been an advisory board member for Amgen, AstraZeneca, Bristol Myers Squibb, Novartis, and Roche. OJ-V has been an advisory board member for AstraZeneca, AbbVie, Boehringer Ingelheim, Bristol Myers Squibb, Merck Sharp & Dohme, and Roche/Genentech. DS has served as an advisory for Roche Canada; he has received grant support from AstraZeneca, Boehringer Ingelheim, Bristol Myers Squibb, Celgene (a Bristol Myers Squibb Company), and Novartis. AA has received honoraria from Boehringer Ingelheim, Bristol Myers Squibb, Eli Lilly, and MSD. RB and TJO are employees of and hold stock in Bristol Myers Squibb Company. MW is contracted for medical review by Celgene (a Bristol Myers Squibb Company) and was employed by Pharmaceutical Research Associates Inc. (PRA) Health Sciences. MR served as a consultant or advisory for AstraZeneca, Boehringer Ingelheim, Bristol Myers Squibb, Celgene (a Bristol Myers Squibb Company), Lilly, Merck Sharp & Dohme, Pfizer, and Roche. DT has been an advisory board member and received honorarium from Celgene (a Bristol Myers Squibb Company). RG serves in an advisory role for AbbVie, AstraZeneca, Baxalta, Boehringer Ingelheim, Celgene (a Bristol Myers Squibb Company), Merck, MSK, Pfizer, and Roche; he serves as a consultant for AbbVie, ARAID, Astellas, Bristol Myers Squibb, Genentech, and INC Research; he has received honoraria from AbbVie. JF was employed by the company Lungenklinik Löwenstein gGmbH. The remaining authors declare that the research was conducted in the absence of any commercial or financial relationships that could be construed as a potential conflict of interest.
